# H7F ameliorates DSS-induced colitis through restoration of intestinal barrier function and inhibition of IL-17/NF-κb signaling

**DOI:** 10.3389/fphar.2026.1853850

**Published:** 2026-07-06

**Authors:** Lili Zhou, Rufei Huang, Jialiang Liang, Wei Sun, Huan Xia, Xinghe Zhou, Xiaolan Zhong, Wanglin Li, Zhaoyang Wang

**Affiliations:** 1 Department of Central Laboratory, Huadu Institute of Medical Sciences, Guangzhou, China; 2 Peptide Source Biotechnology Co., Ltd., Guilin, Guangxi, China; 3 Department of Cell Biology, Jinan University, Guangzhou, China; 4 The First Clinical College of Guilin Medical University, Guilin, Guangxi, China

**Keywords:** gut microbiota, H7F, IL-17, inflammatory bowel disease (IBD), intestinal barrier

## Abstract

**Introduction:**

Inflammatory bowel disease (IBD) is a chronic relapsing disorder of the gastrointestinal tract for which effective and safe therapeutic options remain limited. H7F is an in-house herbal formula developed in our hospital based on clinical practice and traditional Chinese medicine theory. This study aimed to investigate the therapeutic effects of H7F on experimental colitis and to explore the underlying mechanisms.

**Methods:**

Acute colitis was induced in mice by administration of 3% dextran sulfate sodium H7F was orally administered once daily at doses of 100 or 200 mg/kg. Disease severity was evaluated by body weight loss, disease activity index (DAI), colon length, and histopathological changes assessed by hematoxylin and eosin staining. Intestinal barrier function was examined by quantitative real-time PCR (qRT-PCR), Western blotting, and immunohistochemistry for ZO-1, occludin, claudin-1, and Muc2. Inflammatory cytokines (*Tnf-α*, *Il-6*, *Il-10*) and apoptosis-related proteins (Bax and Bcl-2) were also analyzed. In addition, gut microbiota composition was assessed by 16S rRNA sequencing, metabolic alterations by untargeted metabolomics, and transcriptomic changes by RNA sequencing.

**Results:**

H7F treatment significantly attenuated DSS-induced colitis, as evidenced by reduced body weight loss, lower DAI scores, prevention of colon shortening, and alleviated histopathological injury. Mechanistically, H7F restored intestinal barrier integrity by upregulating tight junction proteins and Muc2 expression, suppressed pro-inflammatory cytokines while enhancing *IL10* expression, and reduced colonic epithelial apoptosis by decreasing the Bax/Bcl-2 ratio. H7F also partially reversed gut microbiota dysbiosis, particularly by suppressing pro-inflammatory Peptostreptococcaceae, and corrected metabolic disturbances associated with tyrosine metabolism and ABC transporters. Transcriptomic and molecular analyses further showed that H7F inhibited the IL-17/NF-κB signaling axis, as indicated by reduced IL-17A expression, decreased p65 phosphorylation, and downregulation of *Cxcl-1* and *Cxcl-2*.

**Conclusion:**

H7F ameliorated DSS-induced colitis through restoration of intestinal barrier function, modulation of gut microbiota and metabolism, and inhibition of the IL-17/NF-κB signaling pathway. These findings highlight the therapeutic potential of H7F as a multi-target agent for IBD.

## Graphical Abstract

**Figure:**
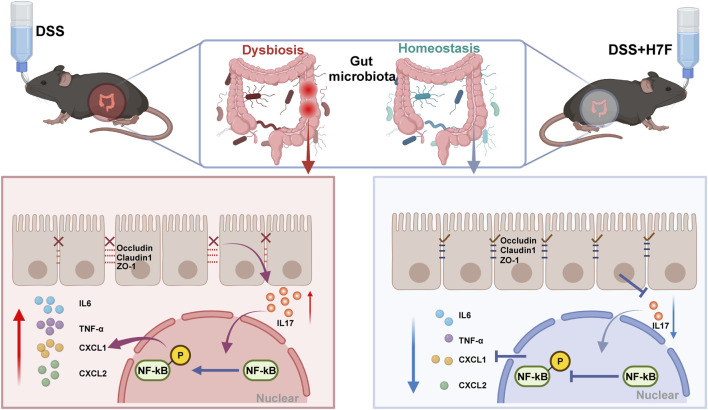

## 1 Introduction

Inflammatory bowel disease (IBD), primarily comprising ulcerative colitis (UC) and Crohn’s disease (CD), is a chronic, relapsing inflammatory disorder of the gastrointestinal tract with increasing global incidence and prevalence (Kaplan and Windsor, 2021; Zhou et al., 2023). The pathogenesis of IBD is multifactorial, involving complex interactions among genetic susceptibility, environmental factors, gut microbiota dysbiosis, and dysregulated immune responses (Friedrich et al., 2019; Cai et al., 2021). Characteristic pathological features include disruption of the intestinal epithelial barrier, excessive infiltration of immune cells, overproduction of pro-inflammatory cytokines (e.g., TNF-α, IL-6, IL-17), and metabolic disturbances (Parikh et al., 2019; Zhao et al., 2021a). Current therapeutic strategies for IBD, such as aminosalicylates, corticosteroids, immunosuppressants, and biologics, aim to control inflammation and induce remission; however, they are often associated with significant side effects, high cost, and a substantial proportion of non-responders or loss of response over time (Magro et al., 2020; Rogler et al., 2021; Gowd et al., 2022). Therefore, the development of safer, more effective, and multi-targeted therapeutic agents remains an urgent clinical need.

Among the multiple inflammatory pathways implicated in IBD, the IL-17/NF-κB signaling axis has attracted particular attention because of its role in linking immune activation to epithelial injury and mucosal inflammation (Li et al., 2025). IL-17A, a signature cytokine mainly produced by Th17 cells, is markedly elevated in inflamed intestinal tissues and contributes to disease progression by promoting neutrophil recruitment, amplifying inflammatory cascades, and aggravating epithelial barrier dysfunction (Miossec and Kolls, 2012; Lee J. Y. et al., 2020). As a central downstream signaling mediator, NF-κB drives the transcription of a broad range of pro-inflammatory cytokines, chemokines, and effector molecules involved in mucosal inflammation, including IL-6, TNF-α, IL-1β, CXCL1, CXCL2, and other inflammatory mediators (Huang et al., 2020; Yuan et al., 2023). Persistent activation of the IL-17/NF-κB signaling axis has been closely associated with excessive immune responses, amplified inflammatory cascades, and sustained intestinal injury in IBD.

Traditional Chinese medicine (TCM) has attracted increasing attention as a potential therapeutic strategy for IBD because its multi-component and multi-target characteristics are well suited to the complex pathogenesis of this disease (Tian et al., 2021; Wang et al., 2024). IBD is driven by intertwined pathological processes, including intestinal barrier disruption, gut microbiota dysbiosis, immune imbalance, and metabolic disturbance, which are difficult to comprehensively control using single-target interventions (Grüner et al., 2023; Pai et al., 2023). Indeed, accumulating evidence has shown that herbal formulas and their bioactive constituents can alleviate experimental colitis by preserving intestinal barrier integrity, regulating gut microbiota composition, correcting metabolic abnormalities, and suppressing excessive inflammatory signaling (Li et al., 2022; Ma et al., 2023). These features make TCM-based interventions especially attractive for IBD management and provide a strong rationale for investigating multi-herb formulas in this setting.

H7F is an in-house herbal formula developed by our hospital based on long-term clinical practice and traditional Chinese medicine theory. In clinical practice, it has been empirically used as a supportive herbal intervention for colitis-related gastrointestinal symptoms, including diarrhea, abdominal discomfort, and impaired mucosal recovery. However, its current application remains largely experience-based, and its therapeutic efficacy and underlying molecular mechanisms have not been systematically evaluated in standardized experimental models.

H7F consists of seven herbal materials: Chu Shizi (*Broussonetia papyrifera* fruit), Mai Ya (germinated *Hordeum vulgare*), Lü Dou (*Vigna radiata*), Bai Ji (*Bletilla striata* tuber), Sha Ren (*Amomum villosum* fruit), Sheng Ma (*Cimicifuga foetida* rhizome), and Shan Nai Zi (*Kaempferia galanga* rhizome). According to TCM theory, these herbs collectively exert effects that strengthen the spleen, resolve dampness, and repair mucosal lesions. Modern pharmacological studies have revealed that individual components of H7F possess anti-inflammatory, antioxidant, and mucosal protective activities. For instance, *B. papyrifera* fruit extract exhibits anti-inflammatory effects by inhibiting NF-κB activation (Lee J. M. et al., 2020; Huang et al., 2025); *B. striata* polysaccharides promote wound healing and epithelial restitution (Zhao et al., 2021b); and *A. villosum* essential oil modulates gut microbiota and alleviates intestinal inflammation (Chen et al., 2018; Gao et al., 2023). In addition, germinated *H. vulgare* contains saccharide and oligosaccharide components that may support gut microbial homeostasis (Peukert et al., 2015); *V. radiata* is rich in polyphenols and other bioactive constituents with antioxidant and anti-inflammatory potential (Hou et al., 2019); *C. foetida* contains Cimicifuga-related triterpenoid glycosides and phenolic acid derivatives with immunomodulatory and anti-inflammatory activities (Guo et al., 2017); and *K. galanga* contains phenylpropanoids and flavonoids associated with anti-inflammatory and gastrointestinal protective effects (Wang et al., 2021). Despite these promising properties, the therapeutic effect of the combined H7F formula on colitis and its underlying mechanisms have not been systematically investigated.

In the present study, we employed a dextran sulfate sodium (DSS)-induced acute colitis mouse model to evaluate the protective effects of H7F. We assessed disease severity, intestinal barrier integrity, inflammatory responses, gut microbiota composition, and metabolic profiles. We further explored the potential molecular mechanisms underlying the anti-colitic effects of H7F, with particular attention to the IL-17/NF-κB signaling pathway.

## 2 Materials and methods

### 2.1 Animals and experimental design

Male C57BL/6 J mice (8 weeks old, 22 ± 2 g) were obtained from the Guangdong Provincial Medical Laboratory Animal Center. All animals were housed under specific pathogen-free conditions with controlled temperature (22 °C ± 2 °C), humidity (50% ± 10%), and a 12-h light/dark cycle, with free access to standard chow and water. After a 1-week acclimatization period, mice were randomly assigned to four groups (n = 8 per group): Control, DSS (model), DSS + low-dose H7F (H7F-L, 100 mg/kg), and DSS + high-dose H7F (H7F-H, 200 mg/kg). The selected doses were not directly converted from the clinical human dose based on body surface area. Since no published dose information is available for the complete H7F formula, 100 and 200 mg/kg were selected as the low and high doses, respectively, based on reported dose ranges of related herbal components in DSS-induced colitis models (Zhu et al., 2022; Choi et al., 2025) and our preliminary dose-range observations at 50–600 mg/kg (data not shown), while avoiding excessive dosing.

Colitis was induced by administering 3% dextran sulfate sodium (DSS, iV, Shanghai, China) in drinking water for seven consecutive days, with fresh DSS solution provided daily (Mo et al., 2022). H7F was administered daily by oral gavage throughout the DSS treatment period, while the Control and DSS groups received an equivalent volume of vehicle (distilled water). Body weight, stool consistency, and rectal bleeding were recorded daily. No anesthesia was used during the experiment, as all procedures were non-invasive. At the end of the experiment, mice were euthanized by carbon dioxide (CO_2_) inhalation followed by cervical dislocation. All animal experiments were approved by the Institutional Animal Care and Use Committee (IACUC) of Jennio Biotech Co., Ltd. (Approval No. K2025-01–190) and conducted in accordance with the ARRIVE guidelines and the 3 R s principles (Percie du Sert et al., 2020).

### 2.2 Chinese medicine formula (TCM)

H7F is an in-house Chinese medicine formula developed by our hospital based on clinical practice and TCM theory. The formula consisted of seven herbal materials: Chu Shizi (*B. papyrifera* fruit), Lü Dou (*V. radiata* seed), Bai Ji (*B. striata*), Sha Ren (*A. villosum*), Sheng Ma (*C. foetida*), and Shan Nai Zi (*K. galanga*) (0.6 g each), together with Mai Ya (germinated *H. vulgare*, 0.15 g). All herbal materials were purchased from Tongrentang Pharmacy (Beijing, China).

The decoction was prepared as follows. The herbal materials were accurately weighed according to the above proportions and placed in a stainless-steel extraction container. Ten volumes of distilled water (v/w) were added, and the mixture was soaked for 30 min. The mixture was first boiled over high heat and then decocted over low heat for 30 min. Subsequently, Bai Ji and Sheng Ma were added to the decoction and further simmered over low heat for 15 min. The decoction was filtered through double-layer gauze, and the filtrate was collected. The residue was then extracted again with eight volumes of distilled water and decocted once more (boiled and then simmered for 30 min). The filtrates from the two extractions were combined and concentrated under reduced pressure using a rotary evaporator at 60 °C–70 °C until the concentration of crude herbs reached 20 mg/mL. The prepared H7F decoction was aliquoted and stored at 4 °C until use. Before oral gavage administration, the decoction was equilibrated to room temperature and mixed thoroughly.

### 2.3 Assessment of colitis severity

Disease activity index (DAI) (Wang et al., 2025) was calculated daily by an investigator blinded to the experimental groups based on three parameters: body weight loss (0: <1%; 1: 1%–5%; 2: 5%–10%; 3: 10%–20%; 4: >20%), stool consistency (0: normal; 1: soft; 2: loose; 3: diarrhea), and rectal bleeding (0: no visible blood; 1: a small amount of blood visible in the feces; 2: a small amount of blood visible around the anus; 3: a large amount of blood visible around the anus). On day 7, mice were euthanized by CO_2_ inhalation followed by cervical dislocation, and colons were harvested. Colon length was measured as an indicator of inflammation, and colon tissues were processed for further analyses.

### 2.4 Histological analysis

Distal colon segments (approximately 1 cm) were fixed in 4% paraformaldehyde for 24 h, embedded in paraffin, and sectioned at 5 μm thickness. For histological evaluation, sections were stained with hematoxylin and eosin (H&E) following standard protocols. Goblet cells were visualized by alcian blue-periodic acid-Schiff (AB-PAS) staining using a commercial kit (G1008, Servicebio, Wuhan, China) according to the manufacturer’s instructions. Stained sections were scanned and analyzed using a slide scanner (Pannoramic MIDI). Histological scoring was performed in a blinded manner based on inflammatory cell infiltration, crypt damage, and mucosal architecture.

### 2.5 Immunohistochemistry (IHC)

Paraffin-embedded colon sections were deparaffinized, rehydrated, and subjected to antigen retrieval in citrate buffer (pH 6.0) at 95 °C for 20 min. Endogenous peroxidase activity was blocked with 3% H_2_O_2_, and non-specific binding was blocked with 5% bovine serum albumin (BSA) in Tris-buffered saline. Sections were incubated overnight at 4 °C with primary antibodies against Muc2 (1:1000, Proteintech, 27675-1-AP), Claudin-1 (1:1000, Proteintech, 28674-1-AP) or Occludin (1:1000, Proteintech, 27260-1-AP). After washing, sections were incubated with HRP-conjugated secondary antibodies, and immunoreactivity was visualized using 3,3′-diaminobenzidine (DAB) substrate. Nuclei were counterstained with hematoxylin. Images were captured using a light microscope (Axio Imager 2, Zeiss, Oberkochen, Germany), and quantitative analysis was performed using ImageJ software (version 1.8.0.345).

### 2.6 Western blot analysis

Colon tissues were homogenized in RIPA lysis buffer (P0013C, Beyotime, Shanghai, China) supplemented with protease and phosphatase inhibitors. Protein concentrations were determined using a BCA assay kit (PC0020, Solarbio, Beijing, China). Equal amounts of protein (30 μg) were separated by 10% SDS-PAGE and transferred onto PVDF membranes (Millipore, Beijing, China). Membranes were blocked with 5% BSA for 1 h at room temperature and incubated overnight at 4 °C with primary antibodies against Claudin-1 (1:2000), Occludin (1:2000), ZO-1 (1:2000, Proteintech, 21773-1-AP), Tnf-α (1:2000, Proteintech, 17590-1-AP), NF-κB (1:2000, Proteintech, 10253-2-AP), phospho-NF-κB (p-NF-κB, 1:2000, Proteintech, 28945-1-AP), Bax (1:2000, Proteintech, 50599-2-Ig), Bcl-2 (1:2000, Proteintech, 60178-1-Ig), Il-17A (1:1000, Proteintech, 13082-1-AP) and β-actin (1:5000, Proteintech, 66009-1-Ig). After washing, membranes were incubated with HRP-conjugated secondary antibodies for 1 h at room temperature. Protein bands were visualized using an enhanced chemiluminescence detection kit (SQ201L, Epizyme, Shanghai, China) and imaged on an E-Blot system (EBLOT, Shanghai, China). Band intensities were quantified using ImageJ and normalized to β-actin.

### 2.7 RNA extraction and quantitative real-time PCR

Total RNA was extracted from colon tissues using TRIzol reagent (ET111-01-V2, TransGen, Beijing, China) according to the manufacturer’s protocol. RNA concentration and purity were assessed using a NanoDrop spectrophotometer (Thermo Fisher Scientific, Waltham, MA, United States of America). Reverse transcription was performed with 1 μg of RNA using a cDNA synthesis kit (AU341-02-V2, TransGen). Quantitative PCR was carried out on a CFX96 Real-Time System (Bio-Rad, Hercules, CA, United States of America) using SYBR Green master mix. The thermal cycling conditions were: 95 °C for 3 min, followed by 40 cycles of 95 °C for 10 s and 60 °C for 30 s. Relative gene expression was calculated using the 2^−ΔΔCt^ method with β-actin as the internal control. Primer sequences are listed in Table 1.

**TABLE 1:** Primer sequences used for qRT-PCR.

Name	Forward primer (5′–3′)	Reverse primer (5′–3′)
*Cxcl-1*	TGG​CTG​GGA​TTC​ACC​TCA​AG	CAA​GCC​TCG​CGA​CCA​TTC​TT
*Cxcl-2*	GCG​GTC​AAA​AAG​TTT​GCC​TTG	AGC​CTT​GCC​TTT​GTT​CAG​TAT​C
*Z O -1*	GCC​TAT​GAA​CCC​CAA​CTT​CCA	TCA​AAC​CGT​AGG​CGA​TGG​TC
*Claudin-1*	TAT​GAC​CCC​TTG​ACC​CCC​AT	AGA​GGT​TGT​TTT​CCG​GGG​AC
*Occludin*	CCT​GAC​CAC​TAT​GAA​ACA​GAC​T	CTC​TTA​TAC​TCC​TGC​AGA​CCT​G
*Tnf-α*	GCT​GTT​GCC​CCT​GGT​TAT​CT	ATG​GAG​TAG​ACT​TCG​GGC​CT
*Il-6*	CTC​CCA​ACA​GAC​CTG​TCT​ATA​C	CCA​TTG​CAC​AAC​TCT​TTT​CTC​A
*Il-10*	TTC​TTT​CAA​ACA​AAG​GAC​CAG​C	GCA​ACC​CAA​GTA​ACC​CTT​AAA​G
*β-actin*	CCC​CTG​AAC​CCT​AAG​GCC​A	CGG​AGT​CCA​TCA​CAA​TGC​CT

### 2.8 16S rRNA gene sequencing and analysis

Fecal samples were collected sterilely on day 7 and immediately stored at −80 °C. Microbial genomic DNA was extracted using the CTAB/SDS method. The V3-V4 region of the 16S rRNA gene was amplified by PCR using specific primers. PCR products were purified using a Qiagen Gel Extraction Kit (Qiagen, Hilden, Germany). Sequencing libraries were generated using the TruSeq® DNA PCR-Free Sample Preparation Kit (Illumina, San Diego, CA, United States of America) and sequenced on the Illumina NovaSeq platform to generate 250 bp paired-end reads. Raw sequencing data were processed using QIIME2 (Version 2020.2). Alpha diversity and beta diversity were calculated. Taxonomic composition was analyzed at the phylum and genus levels.

### 2.9 Untargeted metabolomics

Colon samples (50 mg) were homogenized in ice-cold methanol:water (4:1, v/v) containing internal standards. After vortexing and centrifugation at 12,000 × g for 15 min at 4 °C, the supernatant was collected and dried under nitrogen stream. The residue was reconstituted in acetonitrile:water (1:1, v/v) for Liquid Chromatography-Mass Spectrometry (LC-MS) analysis. Metabolite profiling was performed on an ultra-high-performance liquid chromatography system coupled with a quadrupole time-of-flight mass spectrometer (UHPLC-Q-TOF MS). Chromatographic separation was achieved on a C18 column (2.1 × 100 mm, 1.7 μm) using a gradient elution with mobile phases A (water with 0.1% formic acid) and B (acetonitrile with 0.1% formic acid). Mass spectrometry was conducted in both positive and negative ionization modes. Raw data were processed using Progenesis QI software (Waters, Milford, MA, United States of America) for peak picking, alignment, and annotation against the HMDB, Metlin, and Kyoto Encyclopedia of Genes and Genomes (KEGG) databases. Orthogonal partial least squares discriminant analysis (OPLS-DA) was performed to identify differentially abundant metabolites (VIP >1 and p < 0.05).

### 2.10 Liquid Chromatography-Mass Spectrometry (LC-MS) analysis of H7F

H7F decoction samples prepared by our research team were submitted to Novogene Co., Ltd. (Beijing, China) for LC-MS analysis. Briefly, 2 mL of the H7F decoction was provided for chemical profiling. Sample pretreatment, chromatographic separation, mass spectrometric detection, and primary data processing were performed by the service provider according to their standard operating procedures. The detected compounds were annotated based on mass spectrometric information and comparison with available databases and published reports. Since no reference standards were used for full confirmation in the present study, the identified constituents were considered putatively characterized compounds.

### 2.11 RNA sequencing and bioinformatic analysis

Colon tissue samples were collected from mice by our research team and submitted to Novogene Co., Ltd. (Beijing, China) for transcriptomic analysis. Briefly, approximately 50 mg of colonic tissue from each sample was used for RNA sequencing. Total RNA extraction, RNA quality assessment, library construction, sequencing, and primary bioinformatic analyses were performed by the service provider according to standard procedures. Differentially expressed genes were identified based on the sequencing results, and subsequent functional enrichment analyses, including Gene Ontology (GO) and Kyoto Encyclopedia of Genes and Genomes (KEGG) pathway analyses, were carried out to explore the biological processes and signaling pathways potentially involved in the protective effects of H7F.

### 2.12 Statistical analysis

All data are presented as mean ± standard deviation (SD). Statistical analyses were performed using GraphPad Prism 8.0. Multiple group comparisons were evaluated by one-way analysis of variance (ANOVA) followed by Tukey’s *post hoc* test. A p-value <0.05 was considered statistically significant (*p < 0.05, **p < 0.01, ***p < 0.001, ****p < 0.0001). The sample size for each experiment is indicated in the corresponding figure legend.

## 3 Results

### 3.1 Chemical profiling of H7F by LC-MS

**FIGURE 1:**
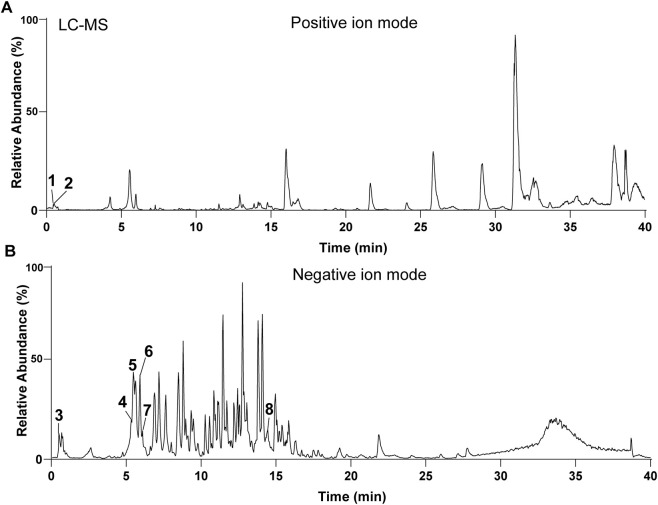
Chemical Profiling of H7F by LC-MS. **(A)** Positive ion mode and **(B)** negative ion mode. The numbered peaks 1–8 correspond to the eight putatively identified compounds listed in Table 2.

LC-MS analysis was performed to characterize the chemical profile of H7F (Figures 1A,B; Table 2). Specifically, the putative Sheng Ma-derived compounds included *2′-acetylacteol glycoside*, *cimicifugic acid E*, and *25-O-methylcimigenol xyloside*, consistent with *Cimicifuga*-related constituents reported previously (Kusano, 2001; Guo et al., 2017). A characteristic phenanthrene glycoside, *2,7-dihydroxy-4-methoxyphenanthrene-2,7-O-diglucoside*, was assigned to *Bai Ji* (Xu et al., 2019). In addition, the compounds associated with *Mai Ya* included maltose and *1F-fructofuranosylnystose*, which are chemically consistent with *saccharide* and *fructooligosaccharide* components reported in barley and germinated barley (Peukert et al., 2015). Several flavonoid glycosides, including *quercetin-7-O-rutinoside* and *kaempferol-7-O-α-L-rhamnoside-3-O-β-D-glucoside*, were chemically consistent with *Chu Shizi* (Chen et al., 2022). The relative percentages were calculated from LC-MS peak responses under the present analytical conditions and should be interpreted as semi-quantitative indicators rather than absolute contents. Although these annotated compounds accounted for a limited proportion of the total ion response, they were retained because they represent characteristic chemical classes and reported bioactive constituents of the corresponding herbs. Together, these findings provide a representative chemical fingerprint of H7F and support subsequent pharmacological investigation and quality evaluation.

**TABLE 2:** Chemical components identified in H7F by LC-MS.

NO.	Compound name	Formula	RT (min)	Ion intensity	Ion mode	Tentative herbal source	Relative percentage (%)
1	Maltose	C_12_H_22_O_11_	0.56	381.0825	[M + H]+	Mai ya	2.337
2	2′-Acetylacteol glycoside	C_31_H_38_O_16_	0.58	705.1789	[M + H]+	Sheng ma	1.283
3	1 F-fructofuranosylnystose	C_30_H_52_O_26_	0.53	873.2721	[M-H]-	Mai ya	0.082
4	Kaempferol-7-O-α-L-rhamnoside-3-O-β-D-glucoside	C_27_H_30_O_15_	5.37	593.1496	[M-H]-	Chu shizi	0.056
5	Quercetin-7-O-rutinoside	C_27_H_30_O_16_	5.56	609.145	[M-H]-	Chu shizi	0.184
6	Cimicifugic acid E	C_21_H_20_O_10_	6	431.0976	[M-H]-	Sheng ma	0.227
7	2,7-Dihydroxy-4-methoxyphenanthrene-2,7-O-diglucoside	C_27_H_32_O_13_	6.52	623.1976	[M-H]-	Bai ji	0.089
8	25-O-Methylcimigenol xyloside	C_36_H_58_O_9_	14.17	633.3998	[M-H]-	Sheng ma	0.103

### 3.2 H7F attenuated DSS-induced colitis in mice

**FIGURE 2:**
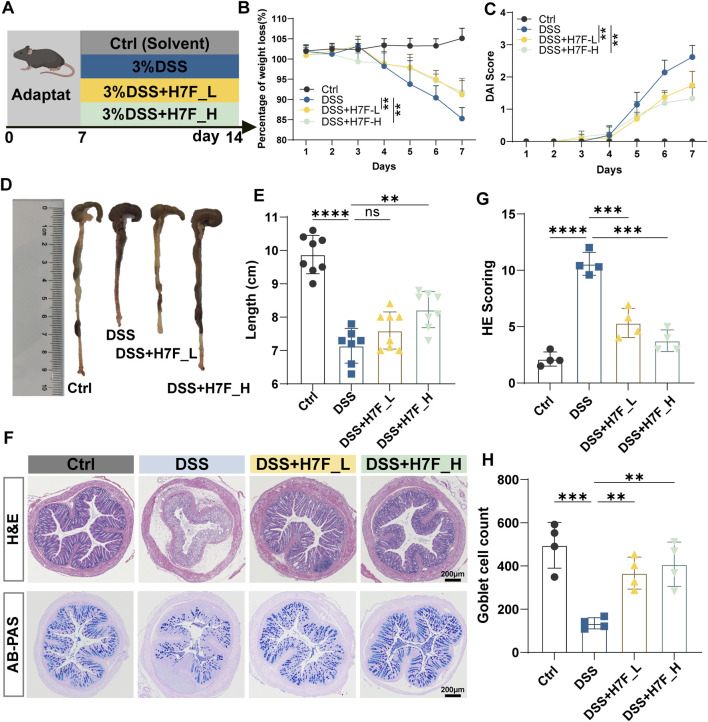
H7F attenuates DSS-induced colitis in mice. **(A)** Schematic diagram of DSS-induced colitis establishment and H7F treatment. **(B)** Body weight changes during the experimental period. **(C)** Disease activity index (DAI) scores. **(D)** Representative images of colons. **(E)** Quantification of colon length (n = 8). **(F)** Representative H&E and AB-PAS staining of colon tissues. **(G)** Histological scores based on H&E staining. **(H)** Quantification of goblet cell numbers based on AB-PAS staining (n = 4). Data are expressed as means ± SD. ns, *p* > 0.05, **p < 0.01, ***p < 0.001, ****p < 0.0001.

To investigate the therapeutic effect of H7F on experimental colitis, mice were administered 3% DSS in drinking water for seven consecutive days to induce acute colitis, while H7F was given by gavage once daily at doses of 100 mg/kg (DSS + H7F-L) or 200 mg/kg (DSS + H7F-H) throughout the experiment (Figure 2A). DSS treatment caused progressive body weight loss from day 4 onward, reaching 14.74% ± 0.33% by day 7. In contrast, the weight loss in the H7F-H treatment group was 8.81% ± 3.46%, while that in the H7F-L group was 8.17% ± 3.39%. Although no significant difference was observed between the two H7F doses, both treatments significantly alleviated DSS-induced weight loss (Figure 2B). Similarly, DSS markedly increased the DAI score from day 4, reaching 2.62 ± 0.36 on day 7, whereas H7F treatment significantly reduced the DAI score to 1.74 ± 0.43 in the low-dose group and 1.33 ± 0.33 in the high-dose group (Figure 2C). Colon shortening, a characteristic feature of DSS-induced colitis, was also evaluated. Colon length was significantly reduced in the DSS group (7.14 ± 0.52 cm) compared with the control group (9.88 ± 0.57 cm). H7F-H significantly restored colon length to 8.22 ± 0.54 cm, whereas H7F-L showed a modest but non-significant improvement (7.60 ± 0.56 cm) (Figures 2D,E). Histopathological examination further confirmed the protective effect of H7F. H&E staining revealed severe mucosal injury in DSS-treated mice, characterized by epithelial disruption, crypt destruction, and marked inflammatory cell infiltration. In contrast, colonic damage was substantially alleviated in both H7F-treated groups, as evidenced by improved epithelial integrity, preserved crypt structure, and reduced inflammatory infiltration. Consistently, histological scores were significantly lower in the H7F-treated groups than in the DSS group (Figures 2F,G). AB-PAS staining further showed a pronounced loss of goblet cells after DSS administration, whereas H7F treatment significantly restored goblet cell abundance (Figures 2F,H). Together, these data indicate that H7F effectively attenuated DSS-induced colitis and protected against colonic mucosal injury.

### 3.3 H7F ameliorated DSS-induced intestinal barrier dysfunction

**FIGURE 3:**
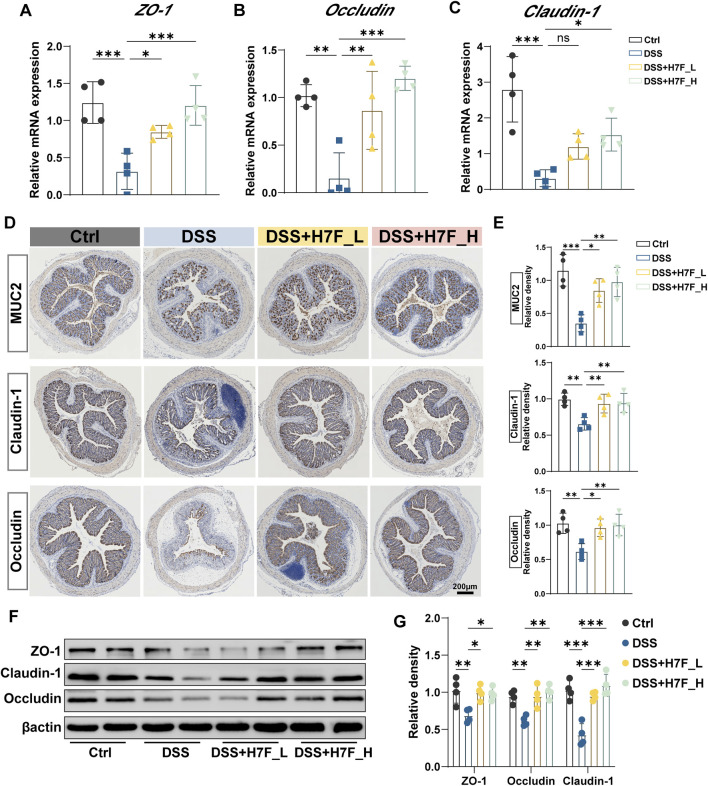
H7F ameliorates DSS-induced intestinal barrier dysfunction.**(A–C)** Quantitative real-time PCR (qRT-PCR) analysis of *Z O -1*, *Occludin*, and *Claudin1* mRNA expression in mouse colon tissues. **(D)** Representative immunohistochemical staining of Muc2, occludin, and claudin-1 in colon tissues. **(E)** Quantitative analysis of immunohistochemical staining shown in **(D)**. **(F)** Western blot analysis of ZO-1, occludin, and claudin-1 protein expression. **(G)** Quantification of protein expression shown in **(F)** (n = 4). Data are expressed as means ± SD. ns, *p* > 0.05, *p < 0.05, **p < 0.01, ***p < 0.001.

To determine whether H7F improved intestinal barrier impairment in DSS-induced colitis, the expression of barrier-related genes was first assessed by qRT-PCR. DSS administration significantly decreased the mRNA levels of *Z O -1*, *Occludin*, and *Claudin-1* compared with the control group. Notably, both low and high-dose H7F treatment markedly restored the expression of these genes (Figures 3A–C). These findings were further validated at the protein level by immunohistochemistry and Western blot analysis. The protein expression levels of Muc2, ZO-1, Occludin, and Claudin-1 were markedly reduced in the DSS group, whereas H7F treatment significantly reversed these changes (Figures 3D–G). These results demonstrate that H7F alleviated DSS-induced intestinal barrier dysfunction and promoted restoration of epithelial barrier integrity.

### 3.4 H7F suppressed DSS-induced inflammatory responses and apoptosis in the colon

**FIGURE 4:**
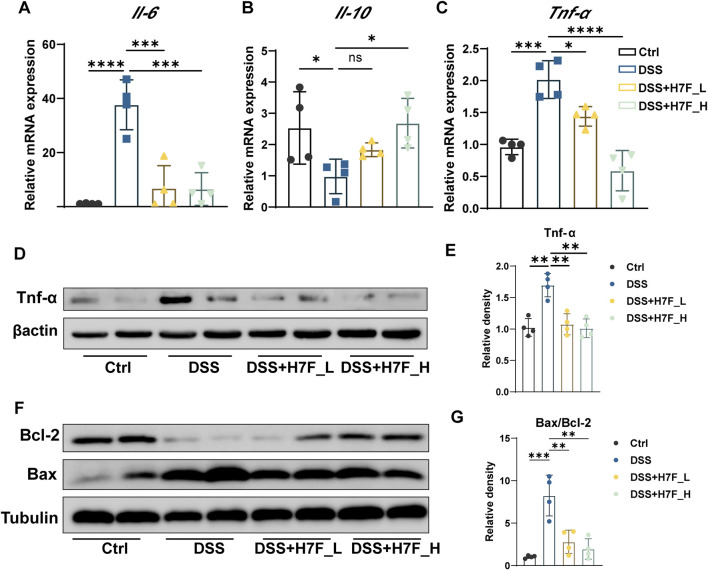
H7F suppressed DSS-induced inflammatory responses and apoptosis in the colon **(A–C)** qRT-PCR analysis of *Il-6*, *Tnf-α*, and *Il-10* mRNA expression. **(D,F)** Western blot analysis of Tnf-α, Bax, and Bcl-2 protein expression. **(E,G)** Quantification of protein expression shown in **(D,F)** (n = 4). Data are expressed as means ± SD. ns, *p* > 0.05, *p < 0.05, **p < 0.01, ***p < 0.001, ****p < 0.0001.

To evaluate the anti-inflammatory effect of H7F, the expression of representative inflammatory cytokines in colonic tissues was examined. DSS treatment significantly upregulated the mRNA expression of the pro-inflammatory cytokines *IL-6* and *Tnf-α*, whereas both doses of H7F markedly suppressed their expression. In contrast, the anti-inflammatory cytokine *IL-10* was significantly downregulated in the DSS group. High-dose H7F significantly restored *IL-10* expression, while low-dose H7F showed an increasing trend without reaching statistical significance (Figures 4A–C). Furthermore, Western blot results showed that H7F was able to inhibit the increase in Tnf-α protein levels induced by DSS (Figures 4D,E), indicating that H7F could alleviate the colonic inflammation caused by DSS.

Beyond inflammation, excessive epithelial apoptosis is another key contributor to colonic mucosal injury in DSS-induced colitis. We therefore examined the expression of the pro-apoptotic protein Bax and the anti-apoptotic protein Bcl-2 by Western blot. The results showed that DSS treatment significantly increased Bax expression, decreased Bcl-2 expression, and consequently elevated the Bax/Bcl-2 ratio compared with the control group (Figures 4F,G), indicating that DSS-induced colitis is associated with robust colonic epithelial apoptosis. Notably, both low- and high-dose H7F treatment significantly reversed these changes, reducing the Bax/Bcl-2 ratio back toward control levels. Collectively, these findings demonstrate that H7F effectively suppressed DSS-induced colonic inflammation and apoptosis, with the high dose showing a relatively stronger effect.

### 3.5 H7F partially reversed DSS-induced gut microbiota dysbiosis

**FIGURE 5:**
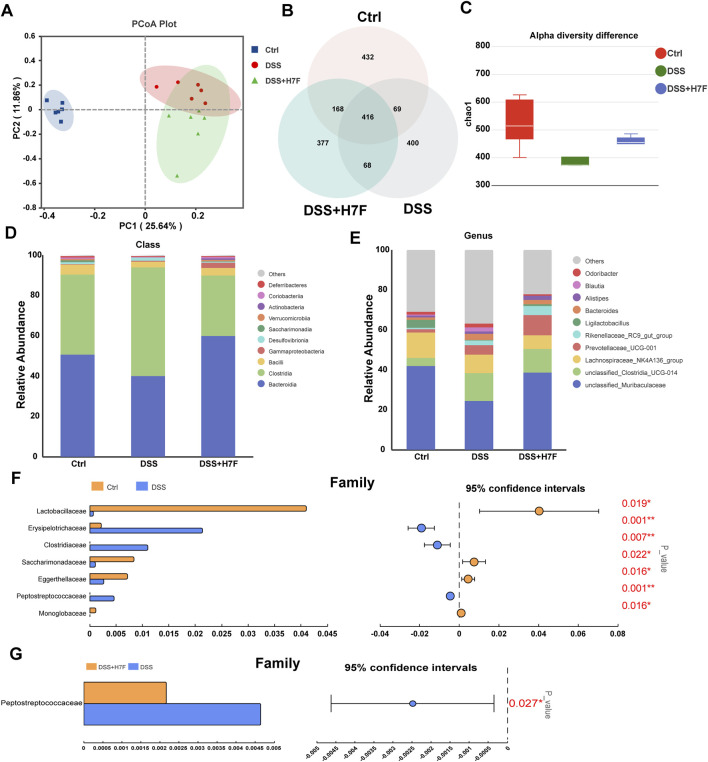
H7F partially restores DSS-induced gut microbiota dysbiosis. **(A)** Principal component analysis (PCA) of gut microbiota based on 16S rRNA sequencing data. **(B)** Venn diagram showing the numbers of shared OTUs among groups. **(C)** Alpha diversity analysis based on the Chao1 index **(D)** Taxonomic analysis at the class level. **(E)** Taxonomic analysis at the family/genus level. **(F,G)** Differential abundance analysis at the family level.

Given the critical role of gut microbiota in the pathogenesis of IBD, 16S rRNA sequencing was performed to assess whether H7F modulated DSS-induced microbial dysbiosis. Principal component analysis (PCA) showed clear separation between the control and DSS groups, indicating that DSS profoundly altered the gut microbial community structure. Although the H7F-treated group remained closer to the DSS group, partial separation from the DSS group was also observed, suggesting that H7F induced detectable remodeling of the microbial composition (Figure 5A). Venn diagram analysis showed that the H7F-treated group shared 584 OTUs with the control group, whereas the DSS group shared only 485 OTUs with the control group, suggesting that H7F partially preserved or restored microbial taxa disrupted by DSS (Figure 5B). In addition, alpha-diversity analysis revealed that the Chao1 index was significantly reduced in DSS-treated mice, while H7F treatment significantly increased this index, indicating recovery of microbial richness (Figure 5C). At the taxonomic level, DSS treatment significantly decreased the abundance of Bacteroidia at the class level and Muribaculaceae at the family/genus level, whereas H7F treatment restored their abundance. By contrast, Clostridia was markedly enriched in the DSS group and reduced after H7F administration (Figures 5D,E). Differential abundance analysis further showed that Peptostreptococcaceae was significantly enriched following DSS treatment, while its abundance was markedly decreased by H7F treatment (Figures 5F,G). These data suggest that H7F partially reversed DSS-induced gut microbiota dysbiosis.

### 3.6 H7F reversed DSS-induced metabolic disturbances

**FIGURE 6:**
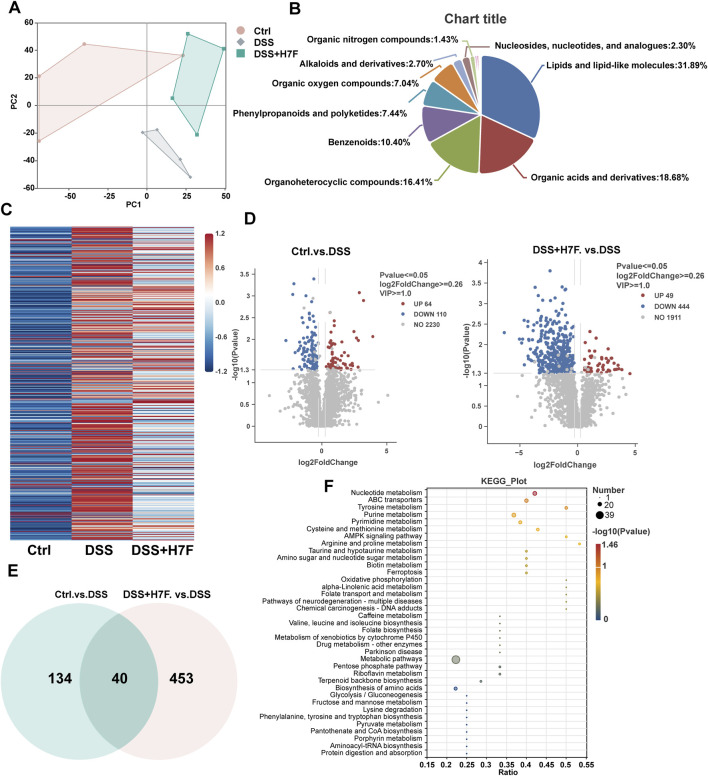
H7F reverses DSS-induced metabolic disturbances. **(A)** PCA of non-targeted metabolomics data. **(B)** Classification analysis of detected metabolites. **(C)** Hierarchical clustering heatmap of metabolites. **(D)** Volcano plots showing differential metabolites. **(E)** Venn diagram analysis of differential metabolites. **(F)** KEGG pathway enrichment analysis of differential metabolites.

To further characterize the effect of H7F on colitis-associated metabolic alterations, non-targeted metabolomic profiling of colon samples was performed. PCA demonstrated clear separation between the DSS and control groups, whereas the H7F-treated group was located closer to the control group, indicating that H7F partially corrected DSS-induced metabolic perturbations (Figure 6A). Classification of the detected metabolites showed that lipids and lipid-like molecules constituted the largest proportion (31.89%), followed by organic acids and derivatives (18.68%) and benzenoids (10.4%) (Figure 6B). Hierarchical clustering analysis further revealed a marked difference in metabolic profiles between the DSS and control groups, whereas the metabolic pattern of the H7F-treated group more closely resembled that of the control group (Figure 6C). Differential metabolite analysis identified 64 upregulated and 110 downregulated metabolites in the DSS group compared with the control group. When compared with the H7F-treated group, the DSS group exhibited 49 upregulated and 444 downregulated metabolites (Figure 6D). Venn analysis identified 40 overlapping differential metabolites between the DSS vs. control and DSS vs. DSS + H7F comparisons (Figure 6E). KEGG enrichment analysis of these shared metabolites showed significant enrichment in tyrosine metabolism and ABC transporters, suggesting that these pathways may contribute to the therapeutic effects of H7F (Figure 6F). Taken together, these results indicate that H7F effectively reversed DSS-induced metabolic disturbances.

### 3.7 H7F may alleviate DSS-induced colitis through inhibition of the IL-17/NF-κb signaling axis

**FIGURE 7:**
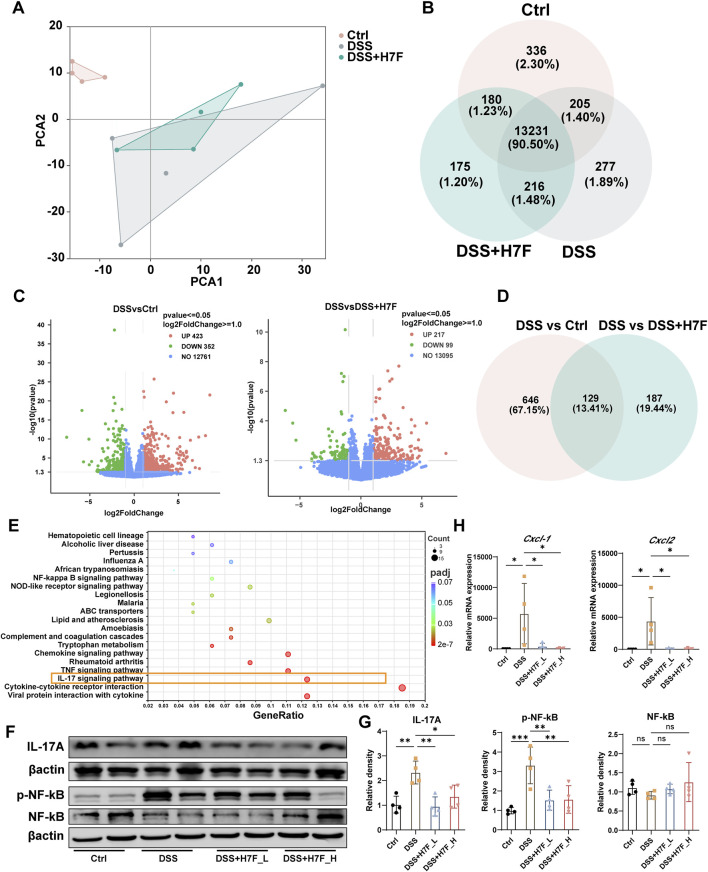
H7F may alleviate DSS-induced colitis through inhibition of the IL-17/NF-κB signaling axis. **(A)** PCA of RNA-seq data. **(B)** Venn diagram showing shared genes. **(C)** Differential gene expression analysis. **(D)** Venn diagram of overlapping differentially expressed genes. **(E)** KEGG pathway enrichment analysis. **(F)** Western blot analysis of IL-17A, total NF-κB, and p- NF-κB protein expression. **(G)** Quantification of protein expression shown in **(F)**. **(H)** qRT-PCR analysis of *Cxcl-1* and *Cxcl-2* mRNA expression (n = 4). Data are expressed as means ± SD. ns, *p* > 0.05, *p < 0.05, **p < 0.01, ***p < 0.001.

To explore the molecular mechanisms underlying the protective effect of H7F, transcriptomic analysis was performed on colonic tissues. PCA showed clear separation of the DSS and H7F-treated groups from the control group. Although the DSS and H7F groups partially overlapped, the H7F-treated samples displayed greater dispersion, suggesting inter-individual variation in the transcriptional response to treatment (Figure 7A). Venn diagram analysis showed that 13,436 genes were shared between the DSS and control groups, whereas 13,411 genes were shared between the H7F-treated and control groups (Figure 7B). Differential expression analysis identified 423 upregulated and 252 downregulated genes in the DSS group relative to the control group. In comparison with the H7F-treated group, 217 genes were upregulated and 99 genes were downregulated in the DSS group (Figure 7C). Among these, 129 differentially expressed genes overlapped between the two comparisons (Figure 7D). KEGG pathway enrichment analysis revealed significant enrichment of the IL-17 signaling pathway among genes altered by H7F treatment, implicating this pathway in the therapeutic action of H7F (Figure 7E). To validate this observation, protein expression of IL-17A, the signature cytokine of the IL-17 pathway, was examined. DSS treatment significantly upregulated IL-17A expression in colon tissues, whereas H7F administration markedly reduced IL-17A levels, indicating that H7F effectively suppresses IL-17A production during colitis. Activation of the NF-κB pathway, a key downstream mediator of IL-17A signaling, was assessed. P65 phosphorylation was significantly increased in the DSS group but markedly reduced after H7F treatment, whereas total p65 levels remained unchanged among groups (Figures 7F,G). In addition, qRT-PCR analysis demonstrated that the downstream target genes CXCL1 and CXCL2 were significantly upregulated by DSS treatment and effectively suppressed by H7F administration (Figure 7H). These findings suggest that H7F alleviated DSS-induced colitis, at least in part, through inhibition of the IL-17/NF-κB signaling axis.

## 4 Discussion

H7F is an in-house Chinese medicine formula developed by our hospital based on clinical practice and TCM theory. In the present study, this hospital-developed formula showed protective effects against DSS-induced colitis, as evidenced by improvements in inflammatory injury, intestinal barrier dysfunction, and gut microbiota-metabolism disturbance.

The pharmacological rationale for the use of H7F may be attributed to the complementary biological activities of its seven herbal components and the representative chemical constituents identified by LC-MS. As shown in Table 2, H7F contained *saccharide*/*fructooligosaccharide*-related components, *flavonoid glycosides*, *phenanthrene glycosides*, *triterpenoid glycosides*, and *Cimicifuga*-related derivatives, which are associated with prebiotic, antioxidant, anti-inflammatory, mucosal protective, and immunomodulatory potential. Specifically, *Chu Shizi* contains flavonoids with anti-inflammatory and NF-κB-modulating potential (Malaník et al., 2020). While the LC-MS-identified *quercetin-7-O-rutinoside* and *kaempferol glycoside* further support its flavonoid-related contribution. *Mai Ya* provides *saccharides* and *fructooligosaccharides* that may support gut microbial homeostasis (Peukert et al., 2015), consistent with the detection of *maltose* and *1F-fructofuranosylnystose*. *Lü Dou* is rich in polyphenols and flavonoids with antioxidant and anti-inflammatory properties (Hou et al., 2019). *Bai Ji* polysaccharides are associated with mucosal repair, wound healing, and intestinal barrier protection (Xu et al., 2019), and the identified phenanthrene glycoside may further reflect its characteristic chemical basis. *Sha Ren* has been reported to modulate gut microbiota and intestinal inflammation (Chen et al., 2018; Xu et al., 2024). *Sheng Ma* contains *triterpenoid glycosides* and *cimicifugic acid* derivatives with immunomodulatory and anti-inflammatory activities (Guo et al., 2017), consistent with the detection of *cimicifugic acid E* and *25-O-methylcimigenol xyloside*. *Shan Nai Zi* contains *bioactive phenylpropanoids* and *flavonoids* with anti-inflammatory and gastrointestinal protective potential (Wang et al., 2021). Therefore, although the pharmacological contribution of each individual herb or compound requires further validation, these herbal components and representative LC-MS-identified constituents together provide a reasonable material and pharmacological basis for evaluating H7F as a multi-component and multi-target intervention for DSS-induced colitis.

Intestinal epithelial barrier dysfunction is a hallmark of IBD, characterized by disruption of tight junction proteins and depletion of mucin-producing goblet cells, leading to increased intestinal permeability (Chen et al., 2021; Qiao et al., 2025). DSS administration significantly downregulated the expression of tight junction proteins (ZO-1, Occludin, Claudin-1) and the secretory mucin Muc2, accompanied by marked goblet cell loss. H7F treatment reversed these alterations at both transcriptional and protein levels. These findings are consistent with previous reports on individual components of H7F. Bletilla striata polysaccharide (BSP), a major bioactive constituent of Bai Ji, has been shown to protect against intestinal epithelial barrier disruption by upregulating ZO-1 and Occludin expression and reducing endotoxin levels in cirrhotic rats (Sun et al., 2025). Polysaccharides derived from *Sha Ren* have also been demonstrated to maintain intestinal barrier function through upregulation of ZO-1 protein expression in colitis mice (Luo et al., 2022). The barrier-protective effects observed with H7F likely reflect the synergistic actions of these herbal components.

Gut microbiota dysbiosis is a critical factor in IBD pathogenesis (Qiu et al., 2022). In our study, 16S rRNA sequencing revealed that DSS treatment profoundly altered the gut microbial community structure, characterized by reduced microbial richness (decreased Chao1 index), depletion of beneficial Bacteroidia and Muribaculaceae, and enrichment of potentially harmful Clostridia. H7F treatment partially reversed these alterations, restoring microbial diversity and taxonomic composition toward control levels. Peptostreptococcaceae, a family within the Clostridia class, was significantly enriched in DSS-treated mice but markedly reduced following H7F administration. Recent evidence has identified *Peptostreptococcus* anaerobius, a member of this family, as a pro-inflammatory bacterium capable of exacerbating DSS-induced colitis through activation of the TLR2/4-NF-κB-NLRP3 axis and induction of macrophage pyroptosis, leading to excessive IL-1β secretion (Guo et al., 2023). The suppression of Peptostreptococcaceae by H7F may therefore contribute to its anti-inflammatory effects. Previous studies have shown that Amomum villosum extracts modulate gut microbiota by increasing short-chain fatty acid (SCFA)-producing bacteria belonging to Firmicutes and Bacteroidetes while decreasing Proteobacteria abundance in TNBS-induced colitis rats (Chen et al., 2018; Xu et al., 2024). The microbiota-regulatory effects of H7F likely result from the combined actions of its polysaccharide-rich components, which can serve as prebiotics to promote beneficial bacterial growth and inhibit pathogenic species.

Metabolic dysregulation is increasingly recognized as a key feature of IBD (Hyun, 2021). In this study, H7F treatment partially reversed DSS-induced metabolic perturbations, with differential metabolites significantly enriched in tyrosine metabolism and ABC transporter pathways. Previous studies have suggested that gut microbial metabolism can influence Th17/IL-17-related immune responses and intestinal inflammation; for example, bacterial metabolic activity can drive Th17 activation and aggravate colitis, while L-tyrosine-derived microbial metabolites such as tyramine and p-cresol may affect epithelial and immune-related functions (Alexander et al., 2022; Blachier and Andriamihaja, 2022; Li et al., 2024). In addition, ABC transporters, particularly ABCB1/P-glycoprotein, are closely involved in epithelial detoxification, barrier maintenance, gut microbiota composition, and susceptibility to intestinal inflammation, and Abcb1a-deficient colitis models have been used to investigate IL-23/IL-17-mediated intestinal immunoregulation (Tanner et al., 2013; Stoeltje et al., 2024). Therefore, combined with our microbiota and transcriptomic data, these findings suggest that H7F may restore microbiota-metabolic homeostasis and thereby contribute to the suppression of intestinal inflammation. However, current evidence does not fully demonstrate that abnormalities in tyrosine metabolism or ABC transporter pathways directly activate the IL-17/NF-κB signaling pathway; thus, these metabolic changes should be interpreted as being associated with, rather than causally upstream of, IL-17/NF-κB pathway activation.

Transcriptomic analysis revealed that the IL-17 signaling pathway was significantly enriched among genes modulated by H7F treatment. The IL-17 family of cytokines, particularly IL-17A produced by Th17 cells, plays a pivotal role in IBD pathogenesis by recruiting neutrophils, enhancing pro-inflammatory cytokine production, and disrupting epithelial barrier function. NF-κB is a key transcription factor downstream of IL-17 and other pro-inflammatory cytokines that drives the expression of inflammatory genes (Miossec and Kolls, 2012; Zhao et al., 2021a). DSS treatment significantly increased p65 phosphorylation and upregulated IL-17 target genes CXCL1 and CXCL2 (Zhang et al., 2022), while H7F administration markedly suppressed these changes. Similarly, the inhibition of the IL-17/NF-κB axis by H7F may involve multiple components. Broussonetia papyrifera (Chu Shizi) constituents have been shown to inhibit NF-κB/AP-1 activation and suppress TNF-α and IL-1β secretion in LPS-stimulated THP-1 cells, and its leaf extract alleviated psoriasis-like inflammation by reducing IL-17A levels and inhibiting the TLR4/NF-κB pathway (Malaník et al., 2020). Amomum villosum extracts have been reported to decrease IL-17 and IFN-γ levels while increasing IL-10 and TGF-β in TNBS-induced colitis rats (Chen et al., 2018). The coordinated inhibition of the IL-17/NF-κB axis by H7F likely represents a central mechanism underlying its anti-inflammatory effects.

The multi-omics approach used in this study enabled us to propose a mechanistic model for H7F’s therapeutic action. Upon DSS-induced epithelial injury, gut microbiota dysbiosis ensues, characterized by depletion of beneficial bacteria (e.g., Bacteroidia, Muribaculaceae) and enrichment of pro-inflammatory taxa (Peptostreptococcaceae). These microbial alterations were accompanied by metabolic disturbances, including alterations in tyrosine metabolism and ABC transporter pathways. Microbial products and damage-associated molecular patterns activate inflammatory signaling cascades, including NF-κB activation, leading to enhanced inflammatory gene transcription and excessive IL-17-associated responses. IL-17 further disrupts tight junction proteins and promotes goblet cell depletion, exacerbating barrier dysfunction and creating a self-perpetuating cycle of inflammation. H7F intervenes at multiple nodes in this pathological network: (1) direct mucosal protection and barrier restoration; (2) modulation of gut microbiota composition, including suppression of pro-inflammatory Peptostreptococcaceae while preserving beneficial taxa; (3) reversal of metabolic disturbances, potentially restoring homeostasis through microbiota-metabolite interactions; (4) inhibition of the IL-17/NF-κB signaling axis, thereby suppressing downstream inflammatory cascades. The multi-target nature of H7F may offer advantages over single-target therapies by addressing the complex, interconnected pathogenesis of IBD.

Several limitations should be acknowledged. First, while we have demonstrated that H7F modulates gut microbiota and inhibits the IL-17/NF-κB axis, the causal relationships between these effects remain to be established. Future studies employing fecal microbiota transplantation or specific pathway inhibitors could help determine whether microbiota changes directly drive the anti-inflammatory effects or *vice versa*. Second, integrated multi-omics correlation analysis was not performed in this study, and the causal links among microbial remodeling, metabolic restoration, and inflammatory pathway inhibition remain to be confirmed. Future studies will use multi-omics correlation analysis and targeted validation experiments to clarify these regulatory relationships. Third, no positive drug control group, such as 5-aminosalicylic acid, mesalazine, or sulfasalazine, was included in the present animal experiment. Therefore, although H7F showed protective effects against DSS-induced colitis, the current data cannot determine whether its efficacy is comparable or superior to that of standard anti-colitis drugs. Future studies including an appropriate positive control are required to further evaluate the relative therapeutic intensity and translational potential of H7F.

## 5 Conclusion

In conclusion, H7F attenuates DSS-induced colitis by restoring intestinal barrier function and inhibiting the IL-17/NF-κB signaling axis. To our knowledge, this is the first study to apply a multi-omics approach to systematically evaluate the therapeutic mechanism of H7F, revealing that its anti-colitic effects involve coordinated regulation of gut microbiota, metabolic pathways, and the IL-17-driven inflammatory cascade. These findings establish H7F as a multi-target candidate for IBD and provide a scientific basis for its future clinical translation as a complementary therapy.

## Data Availability

The original contributions presented in the study are publicly available. This data can be found here: https://www.ncbi.nlm.nih.gov/bioproject/PRJNA1481026 and https://www.ncbi.nlm.nih.gov/bioproject/PRJNA1481124. The metabolomics data (LC–MS peak intensity tables) are included in the Supplementary Material. Further inquiries can be directed to the corresponding authors.
