# Supplementary material for: H7F ameliorates DSS-induced colitis through restoration of intestinal barrier function and inhibition of IL-17/NF-κb signaling
Source: Front Pharmacol. 2026 Jul 6;17:1853850. doi: 10.3389/fphar.2026.1853850 (PMC13382494; doi:10.3389/fphar.2026.1853850)

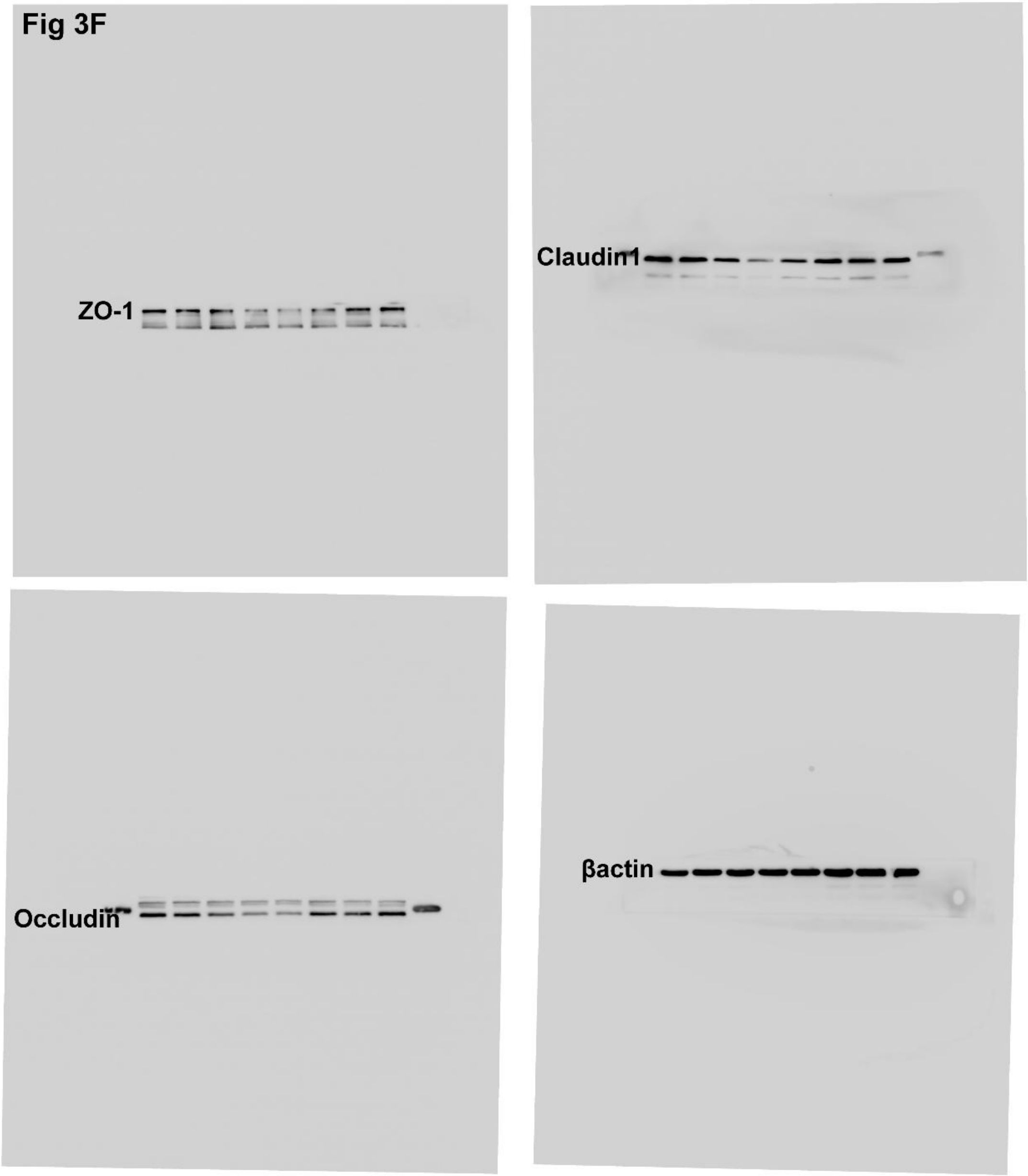
Fig 3F
ZO-1
Claudin1
Occludin
βactin

Fig 4D

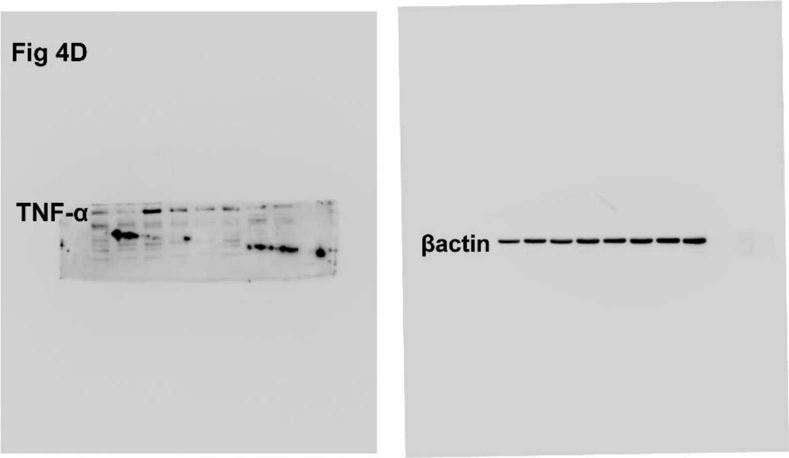

Fig 4F

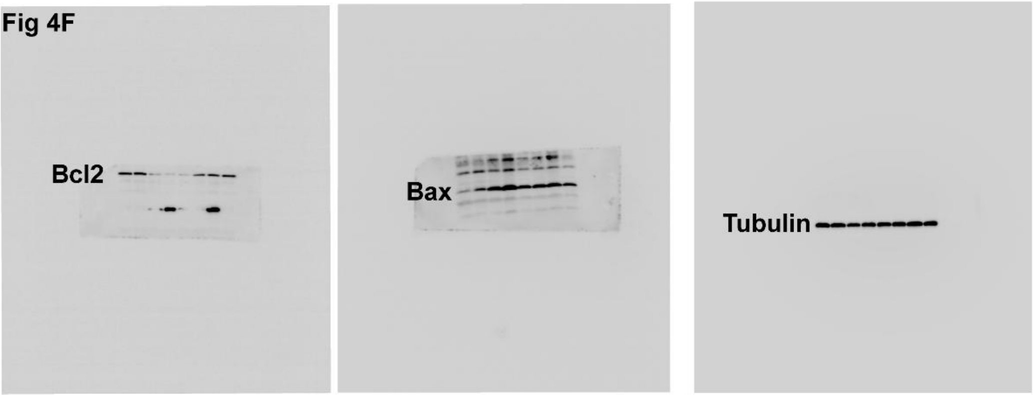

Fig 7F

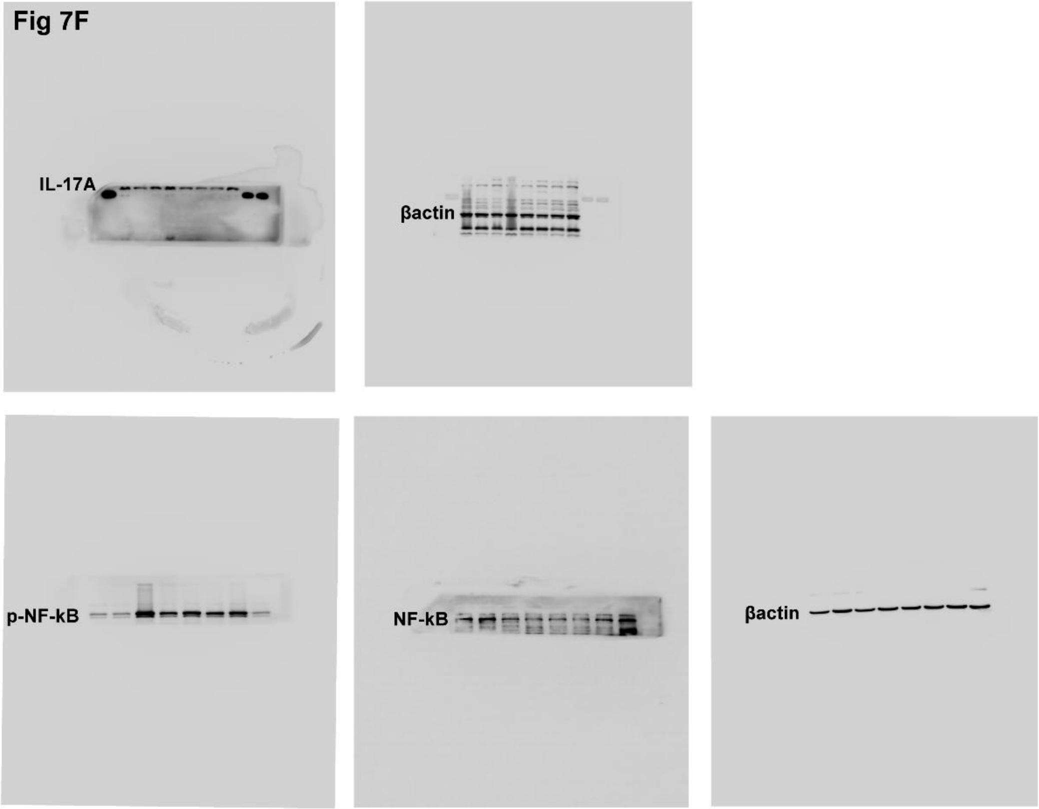

Supplement: Supplementary file 1 [file Supplementaryfile1.pdf]
